# Eye care for the people by the people: a case study from the Sundarbans

**Published:** 2020-12-31

**Authors:** Asim Sil

**Affiliations:** 1 Medical Director: Netra Niramay Niketan, West Bengal, India.

**Eye care service delivery in the Sundarbans of Bengal is an inspiring story in which concerted efforts involving training and participation of local resources led to a significant reduction of avoidable blindness.**

**Figure:**
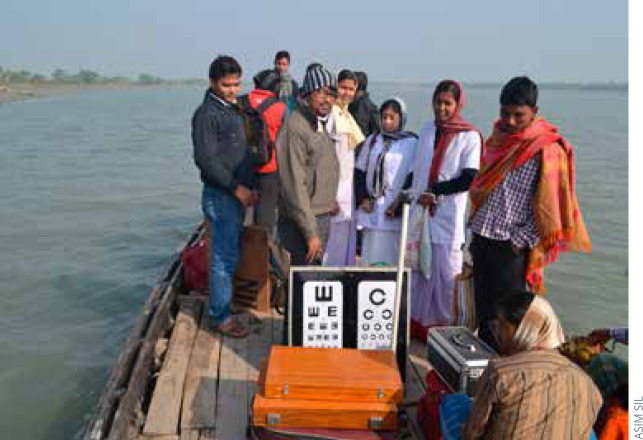
Reaching out to the islands. **SUNDARBANS**

The Sundarbans is a unique geographic area that is home to the world's largest mangrove forest and the Royal Bengal Tiger. Spread along the border between India and Bangladesh, the Indian section of the Sundarbans falls within the districts of North 24 Paraganas and South 24 Paraganas in West Bengal and consists of 106 islands; 52 of which are inhabited. Most islands are accessible by boat only, which makes life very challenging for the 4.7 million people who live in the Sundarbans. Nearly half of the population (47%) belong to the most marginalised groups in India: the Scheduled Casts and Scheduled Tribes. Over 40% of households live below the poverty line, and 13% come under the category of the ‘poorest of the poor’.^1^

Extreme levels of poverty and the challenging terrain limit access to health care and education. Many people are migrating to cities and towns for work due to shortage of job opportunities in the region. The insufficient number of health care providers is further adding to the woes of the Sundarbans.

## Organising eye care in the Sundarbans

Any region with inherent geographical access barriers and socio-economic deprivation faces immense challenges in health care delivery. The Sundarbans is no exception in this context. However, the island network had an opportunity to improve this situation when Standard Chartered Bank came forward to support eye care services in the region. As a part of its ‘Seeing is Believing’ initiative, the bank supported the Sightsavers to implement, ‘Sundarbans Eye Health Service Strengthening Project’ in West Bengal. The objective of the five-year project, 2014-2018, was to contribute to the elimination of avoidable blindness in the area. Sightsavers partnered with the government and three non-government organisations to implement the project.

## Developing human resources

The core strategy of the initiative was to use locally available human resources to strengthen eye health services in the region. This was important because health professionals from outside the region were less likely to stay in those difficult conditions. The initial plan was to appoint qualified optometrists from outside the Sundarbans, but after analysing the ground realities, local young people were selected for training as vision technicians (VTs) and community health workers (CHWs).

The main challenge was the availability of suitable local candidates for the job. The local social organisations played an important role in identifying suitable candidates. The minimum eligibility for the position of VTs and CHW was high school class 12 and class 10, respectively. The total training period for VTs was one year; for CHWs, it was three months. A recognised training institute, a partner hospital, imparted this training.

The training covered awareness of common eye diseases and eye care workers' role in the management. It also covered counselling and outreach work. The trainers ensured that the VTs were well trained in retinoscopy for refraction. As a part of the project a baseline population based survey was conducted to assess the magnitude of blindness and visual impairment. All the newly trained VTs and CHWs participated in conducting the fieldwork. This helped them in understanding the ground realities of the service area.

Six months after the start of the program, more candidates had to be recruited and trained, as some candidates had left before completing their course.

**Table 1:** Baseline survey results at the start of project (in 2014) and in 2018, five years after the vision centres became operational.

Indicators	2014 baseline values	2018 end line values
**Prevalence of blindness bilateral best-corrected vision (age and sex adjusted)**	Aged 40+: 1.5%	Aged 40+: 0.7%
	Aged 50+: 2.4%	Aged 50+: 1.3%
**Cataract surgical coverage (person, in sample)**	3/60: 75.0%	3/60: 86.3%
	6/60: 49.6%	6/60: 55.5%
**The extent of coverage of eye health services within the project area (access to near glasses)**	46.2%	60.3%

## Service activities

In total, 17 VTs were trained and then deployed to 17 vision centres in the region that had been established as part of the project. The VTs performed refraction and provided spectacles at an affordable cost. They also detected cataract and other conditions, and referred patients to hospitals run by the government or by non-governmental organisations. These centres provided spectacles to beneficiaries at an affordable cost. CHWs assisted the VTs in the mission.

An ophthalmologist from the base hospital visited the vision centres once a month to attend to the referral patients and support the VTs and CHWs in various activities such as refining clinical skills, meeting local community etc. The VTs and CHWs also raised awareness of eye health by talking to people and offering one-to-one counselling and health education. Over some time, they came to be seen as a reliable source of information for the local community.

The hospitals organised eye screening camps in the remote villages inside the islands of Sundarbans, and held school eye examinations near the vision centres with the help of VTs and CHWs. The CHWs distributed free spectacles to children who were identified through school screening and refracted at the vision centres. People who needed cataract operations were taken to the base hospital, in groups by hospital vehicles, and the follow-up was arranged at the vision centre. This entire service was offered free of cost to patients.^2^

## Training other health care workers

Training other groups of health care providers was a significant component of the project. The group included 1,467 accredited social health activists (ASHAs) and auxiliary nurse midwives (ANMs) who operate at the grassroots level in the government health system. Also 2,380 rural medical practitioners (RMPs) were included. RMPs are important health providers in remote areas. 3,585 health ambassadors, 70 paramedical ophthalmic staff, and 2,349 school teachers were given orientation programme in primary eye care. These personnel, in turn, worked closely with VTs and CHWs.

## Assessing the impact - comparing baseline with the endline survey results

A population-based eye health survey was conducted in the Sundarbans in 2014, at the start of the project, to assess eye health status and health-seeking behaviour of the people in the region. The participants were individuals aged 40 years and above. As part of the survey, it was found that cataract and uncorrected refractive error were the leading causes of blindness and visual impairment. About 75.2 per cent of the sample had presbyopia, while less than half (46.2 per cent) had access to near vision spectacles. Of the remaining population, more than half (54 per cent) did not even realise that they needed spectacles.^3^ These findings played an important role in strategic planning. The population-based survey was repeated in the same area, towards the end of the project in 2018, applying the same methodology. The key findings are summarised in table 1.

The results of the study indicate good progress in eye care in the Sundarbans, with improved coverage and quality of eye health services. Gender differences were not statistically significant.^4^

## Conclusion

For reaching out to the people residing in difficult geographical terrains, the local human resources are always the best choice to engage, in terms of their acceptance and effectiveness. Moreover, proper selection of candidates, good training and support can bring more success. The VTs are the face of the hospital or any programme in the community. The commitment levels of the VC staff and the quality of their services determine the success of the respective units. Retaining trained and efficient human resources continues to be a big challenge. The effective strategy lies in conducting continuous training for VTs to enhance their competencies and refining institutional policies in favour of retention. Periodic visits by senior staff from the base hospital can strengthen the relationship as well as improve the quality of service. The capacity building of local people indeed brings a sustainable change in health care. After the completion of the project the vision centres are being run by the local eye health workers under the management of respective partner hospitals.
